# Effects of variable resistance training within complex training on strength and punch performance in elite amateur boxers

**DOI:** 10.3389/fphys.2024.1472258

**Published:** 2024-10-21

**Authors:** Yongfu Liu, Zijing Huang, Zixiang Zhou, Liqin Zhang, Yuqiang Guo, Chao Chen

**Affiliations:** ^1^ School of Athletic Performance, Shanghai University of Sport, Shanghai, China; ^2^ Inner Mongolia Institute of Sport Science, Hohhot, China; ^3^ College of Physical Education, Dalian University, Dalian, Liaoning, China

**Keywords:** variable resistance, complex training, muscle strength, punch ability, athletic performance

## Abstract

**Objectives:**

This study explored the effects of 6 weeks of variable resistance training (VRT) and constant resistance training (CRT) within complex training, on muscle strength and punch performance.

**Methods:**

Twenty-four elite female boxers from the China National team were divided randomly between an experimental group (VRT) and a control group (CRT). Maximum strength of the upper and lower limbs, countermovement jump (CMJ) performance, and punch performance (single, 10s and 30s continuous) were assessed pre- and post- intervention.

**Results:**

VRT and CRT showed significant increases (*p* < 0.001) in the bench press (ES = 1.79 and 1.07, respectively), squat (ES = 1.77 and 1.10, respectively), and CMJ (ES = 1.13 and 0.75, respectively). The bench press (*p* < 0.05) and squat (*p* < 0.05) improved significantly more following VRT compared to CRT. Additionally, single punch performance (speed, force, and power) increased significantly in the experimental group (ES = 1.17–1.79) and in the control group (ES = 0.58–1.32), except for the lead punch force in the control group (*p* > 0.05, ES = 0.20). 10s continuous punch performance (number, speed, force, and power) increased significantly (both *p* < 0.05) in the experimental group (ES = 0.52–1.65) and in the control group (ES = 0.32–0.81). 30s continuous punch performance (number, force, and power) increased significantly increased significantly (both *p* < 0.05). However, no statistically significant differences were found between groups for punch performance.

**Conclusion:**

These findings provide evidence that VRT may improve maximum muscle strength in both upper and lower limbs, vertical jump and punch performance in elite amateur boxers.

## Introduction

Complex training is the most used format for physical performance enhancement by athletes (Zhong et al., 2023). His approach combines various forms of traditional resistance training with plyometric training, demonstrating its effectiveness in improving multiple physical health outcomes, including straight sprint speed, vertical jump height, and change of direction speed (CODS) (Thapa et al., 2024). The resistance training component of complex training primarily involves constant resistance, which can enhance athletic strength and power (Frost et al., 2010). In constant resistance training (CRT), the external load is typically maintained consistently throughout the range of motion. This approach can lead to the emergence of the “sticking point” phenomenon, which is commonly observed in resistance training (Kompf and Arandjelović, 2016). The presence of a sticking point decreases the speed during the second half of the resistance movement (Haff, 2016), leading to an inconsistent magnitude of mechanical stimulus throughout the range of motion (Andersen et al., 2022). Variable resistance training (VRT) refers to methods that combine iron chains, elastic bands, and free-weights to enhance both maximum and explosive strengths (Heelas et al., 2021). It could be argued that using variable resistance would shorten the deceleration phase and hence increase the barbell velocity and mean power throughout the movement (Andersen et al., 2022). Previous studies have shown that VRT significantly increases neuromuscular activation, recruiting more muscle fibers and thus improving maximum strengths compared with CRT (Andersen et al., 2016). A number of studies have examined acute neuromuscular responses during VRT and CRT, and most studies confirmed that VRT was superior to CRT in terms of improving muscle strength (Andersen et al., 2020; Kubo et al., 2018).

Boxers throwing a punch is a complex process that involves the force generated by the lower limbs pushing off the ground, the rotation of the trunk, and the extension of the upper limbs (Yi et al., 2022). The striking power of punches is crucial for a boxer’s success in a match (Beattie and Ruddock, 2022), and punch impact depends on strength and power (Yi et al., 2022). It depends on the strength of the muscles in the upper and lower limbs (Dunn et al., 2022). Strength training is an effective way to directly and significantly improve the athletic performance of boxers. Well-developed strength and explosiveness can effectively enhance the effectiveness of punches (Beattie and Ruddock, 2022). Additionally, a boxer’s level of explosiveness, coordination, reaction, and other abilities are closely related to their strength qualities. Effective strength training not only promotes a boxer’s understanding of technique but also improves their ability to control and dominate matches (Chen et al., 2018). Moreover, few studies have focused on the use of VRT to improve muscle strength and punching performance in boxers.

Therefore, this study incorporated variable resistance into compound training, to explore the effect of variable- and constant- resistance training within complex training on boxers’ muscle strength and punch performance, specifically exploring its effects on maximum upper, lower limb strength and punch performance. We hypothesized that VRT and CRT would lead to increases in maximum muscle strength and punching performance, with VRT demonstrating a greater improvement compared to CRT.

## Methods

### Participants

**FIGURE 1:**
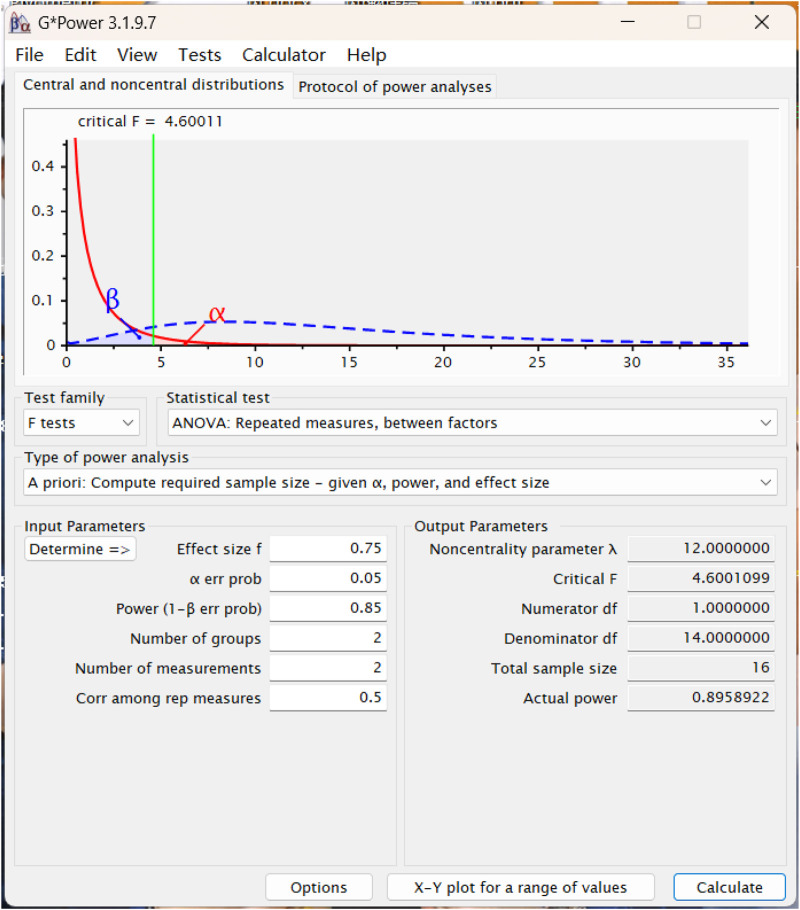
Detail of sample size calculation.

A total sample size of at least 16 participants was determined with the use of G-power3.1 following a power calculation for 85% statistical power, an alpha error of 0.05 and an effect size of 0.75 (Arazi et al., 2020) (Figure 1). All elite female boxers from the China national team volunteered to participate in the study, totaling 24.

All participants had more than 2 years of resistance training experience. The participants were stratified randomization into VRT (n = 12) and CRT (n = 12) groups according to competition kilogram class (Table 1). All participants were informed of the experimental procedures and signed informed consent. Ethical consent was provided by the Shanghai University of Sport Research Ethics Committee (approval number:102772021RT029) and in accordance with the Helsinki declaration.

**TABLE 1:** Participant characteristics of study participants.

Group	Heigh (cm)	Body weight (kg)	Age (yrs)	Training experience (yrs)
VRT	170.55 ± 7.78	61.73 ± 8.52	25.74 ± 3.52	9.82 ± 2.48
CRT	171.24 ± 6.23	62.60 ± 7.61	24.87 ± 2.89	8.73 ± 2.66

Values are given as mean ± *SD*.

### Experimental design

A mixed design exploring both within- and between-groups differences was used to compare the effects of VRT and CRT within a complex training program on maximum strength and punch performance. VRT and CRT were based on boxing-specific technical movements to improve strength and explosive power, and thereby enhance punch performance. From February 2023 to April 2023, each player completed resistance training sessions for 5 weeks (2 sessions/week), and each interval between each complex training session was at least 48 h (Haff, 2016). All testing and training sessions took place at the same venue under the direct supervision of the lead investigator. Pre- and post- testing, ensure that each test is scheduled at the same time of day.

One week before the baseline test, the participants started to become familiar with the test procedures, intervention, and team heart rate band (Polar Team Pro, Finland). After familiarizing, the players were randomly assigned into two groups using the SPSS random number generator based on weight class: CRT (n = 12) and VRT (n = 12).

### Intervention protocol

The participants trained twice weekly for a total of 12 sessions of 100 min each. Training included seven boxing-related movements, centered on lower limb and trunk rotations and upper limb ballistic movements (Table 2). The protocol was conducted with 2∼5 exercise sets (e.g., bench press, medicine ball toss, drop jump), with each set including 1∼5 resistance exercises and 5∼15 plyometric exercises (Ebben, 2002).

**TABLE 2:** Details of the VRT program.

Exercises	Intensity	Repetitions (number) (s)	Training purpose
Bench press (elastic band) + barbell flat push	85%1RM + 5/10 kg	5 + 6 + 7	Upper limb explosive strength
Deep squat (iron chain) + CMJ	85%1RM + body weight	5 + 6 + 7	Lower limb explosive strength
Knee barbell high pull (iron chain) + box jump	85%1RM + body weight	5 + 6 + 7	Whole body explosive strength
Barbell cannon rotating push (elastic band) + solid ball rotation	85%1RM + 4 kg	5 + 6 + 7	Rotating explosive strength
Barbell lunge (iron chain) + lunge jump	85%1RM + body weight	5 + 6 + 7	Lower limb explosive strength
Bench pull (iron chain) + dumbbell back flight	85%1RM + 15/20 kg	5 + 6 + 7	Back explosive strength
Hip thrust (iron chain) +push solid ball in a kneeling position	85%1RM + 4 kg	5 + 6+ 7	Trunk explosive strength

RM: repetition maximum; CMJ: countermovement jump.

The total duration of each training session was 100 min, including 20 min for special resistance preparation, 60 min for the main body of variable resistance compound training, and 20 min for recovery and regeneration. The selection of training methods is based on the technical characteristics of boxing, prioritizing power chain movements that are highly correlated with boxing ability. From the primary to the secondary, the three main force generation segments of the lower limbs pushing off the ground, trunk rotation and transmission, and upper limb end output are respectively carried out. Finally, seven compound training movements of variable resistance are determined (Table 2).

**FIGURE 2:**
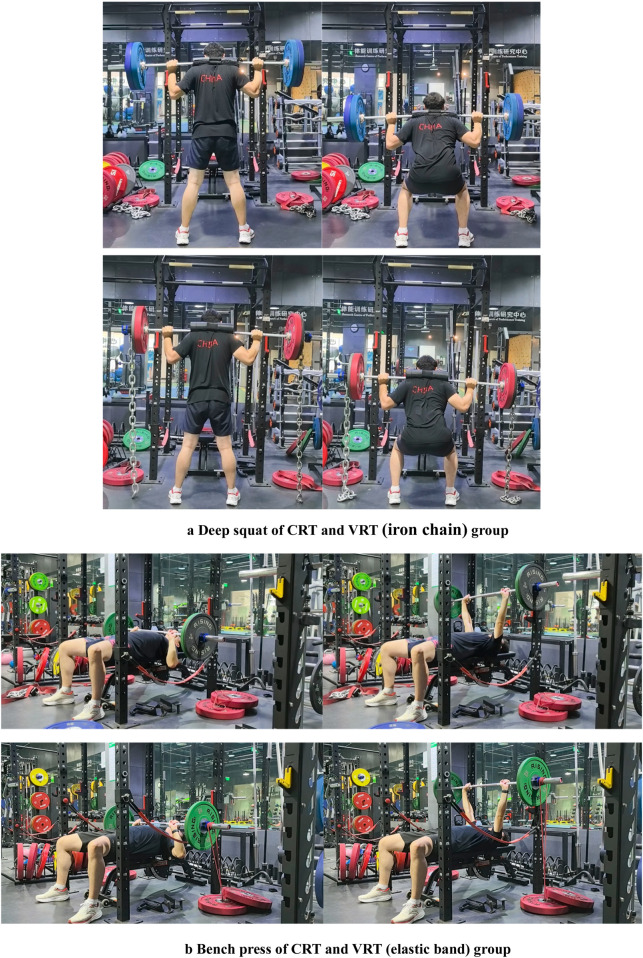
**(A)** Deep squat of CRT and VRT (iron chain) group. **(B)** Bench press of CRT and VRT (elastic band) group.

The training load for variable resistance compound training was organized based on the principle of compound training (post-activation enhancement effect). Each exercise consisted of 1∼5 sessions of resistance training and 5 ∼ 15 sessions of combined rapid stretching training (Ebben, 2002). Resistance training was performed at 85% intensity, while rapid stretching combined training was conducted at 75% intensity. In VRT, variable resistance accounts for 15%–20% of the total resistance, while constant resistance accounts for 80%–85% (Andersen et al., 2015; Bellar et al., 2011)of the total resistance. In CRT, constant resistance accounts for 100%. In order to fully benefit from the post-activation potentiation effect induced by high-load resistance training (Shi et al., 2022c), it is essential to complete the conversion of explosive power (both general and specific) within 30s after the resistance training session (Figures 2A, B). Among them, the first 15s were dedicated to active recovery, while the last 15s involved six rapid stretching compound training followed by 7s consecutive of explosive air punches. The action interval was 2 min, and the intergroup interval was 4 min (Suchomel et al., 2018).

The maximum strength level of athletes increased steadily with the progress of physical training. The stability of weight-bearing exercises such as bench press, squat, high pull, barbell rotation press, barbell lunge, bench pull, and hip jerk increased by 10.32%, 16.55%, 7.68%, 9.35%, 8.35%, and 18.25%, respectively. During the two training sessions last week, the load was reduced by 40% due to the proximity of the competition. This reduction involved decreasing the number of sets to three, while keeping other training elements unchanged.

### Testing procedures

**FIGURE 3:**
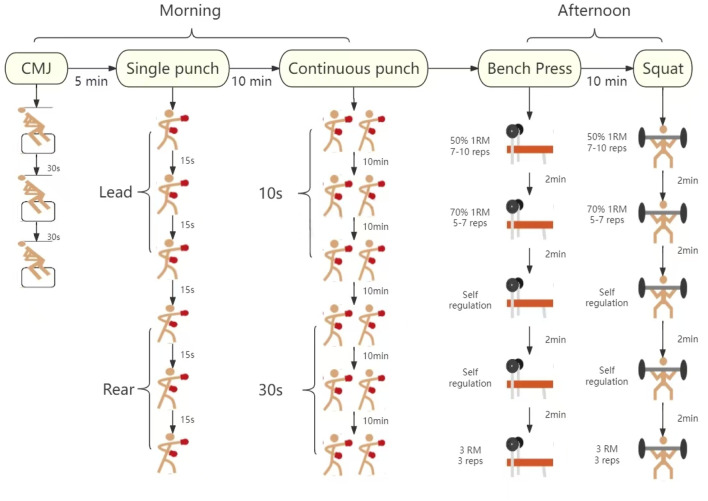
Schematic diagram of the test process.

Testing involved evaluating strength and specialized punching ability. All tests were completed in 1 day, which was 3 days before and after the training protocol. The strength indexes included the relative strength of bench press and squat (kg), and the CMJ (cm). The special punching ability indexes include: (1) single punching ability, which comprises the maximum hitting speed (m/s), relative maximum hitting power (kg) and relative maximum hitting power (w) of the front and back straight punches; (2) continuous punching ability, which involves the number of punches (NP, numbers), average punch speed (APS, m/s), relative cumulative punch force (CPF, kg), and relative cumulative punch power (CPP, w) during 10s and 30s of consecutive punching (Figure 3).

In order to evaluate the punching ability of boxers at different levels more effectively and objectively, the following calculation methods are used in the study when the indexes related to punch force and power are involved: relative cumulative punch force = cumulative punch force/body weight; relative cumulative punch power = cumulative punch power/body weight.

### Outcome measures

#### Three repetition bench press and squat test

After warming up, based on the participant’s body weight and training experience, a weight of 50% of 1RM is used to perform 7–10 repetitions, followed by a weight of 70% of 1RM for 5–7 repetitions. Then, the weight is increased according to the participant’s condition until the participant can only perform the weight for three repetitions. There is a 2-min interval between each set. Both bench press and squat tests are completed within five sets. 1RM was estimated based on the relation of % RM-repetitio as determined by the National Strength and Conditioning (Haff, 2016).

#### Countermovement jump

The jump mat (Smart Jump; Fusion Sport, Coopers Plains, Australia) was used for the CMJ test. Participants were asked to dip to a self-selected depth before jumping vertically with hands on their hips, then performed a rapid downward movement of self-determined depth, followed by a vertical maximal height leap while maintaining straight legs. Based on prior studies (Markovic et al., 2004), three trials with a 30s recovery period were conducted for each jump, and the best performance was used for analysis. Before each jump test, there were two submaximal practice trials with a 1-min recovery period.

#### Punch performance test

The boxing training assistant system (Xingxun, NY-BX101, China) was used to test participants’ punch performance. Punching performance included single punch (punch speed, punch force, punch power) and continuous punch (punch times within 10s and 30s, cumulative punch force, and cumulative punching power). The punch bag (Jiurishan, China) was a suspended microfiber sandbag of 35 cm diameter and weighing 40–45 kg. For the single punch test, participants were instructed to step forward and use maximum effort. Prior to the test, participants performed submaximal attempts at lead and rear straight punch. According to the previous protocol (Loturco et al., 2016), this test was conducted with three lead straights and three rear straight punches from a self-selected position, and the best attempt was used in the analysis. A 15s rest was provided between each punch. The continuous punching test required alternating lead- and rear-hand straight punches to a fixed point without interruption from a self-selected position. The test was repeated three times with a minimum of 10 min of rest between each test. The self-selected position was determined by each participant to elicit best performance. The participants were instructed to give “all-out” effort during continuous punching test and were verbally encouraged throughout the duration of the workout to maintain a maximum effort.

### Statistical analysis

Descriptive data are presented as mean (M) ± standard deviation (SD). The Shapiro–Wilk test was used to examine normality of distribution. A 2 (groups: VRT and CRT) × 2 (time: pre-intervention and post-intervention) repeated-measures ANOVA with Bonferroni adjustment was used to evaluate each variable using the mean difference to examine group × time interactions and within- and between-group differences. For the differences, the 95% confidence interval and the percentage of change are calculated. Effect sizes (ES) were calculated using Cohen’s d, interpreted as minimal (<0.2), small (0.2–0.5), moderate (0.5–0.8), or large (>0.8) (Cohen, 1988). Statistical analyses were conducted using SPSS version 25.0 (IBM, Armonk, NY, United States).

## Results

### Strength performance

The mean values and changes in the muscle strength performance measurement are showed in Table 3. Significant time and group interaction (*p* < 0.001, η_*p*_^2^ = 0.637) were observed for the bench press test. Simple effects analyses revealed significant post-experiment improvement in the VRT (*p* < 0.001, CI = 0.140 to 0.184, ES = 1.79) and the CRT (*p* < 0.001, CI = 0.046 to 0.090, ES = 1.07), and a significant between-groups post-experiment difference (*p* = 0.007, ES = 1.33). For squat performance, there was a significant time by group interaction (*p* = 0.002, η_*p*_^2^ = 0.369). The squat performance improved significantly more following VRT compared to CRT (*p* = 0.021, ES = 0.96). Both groups showed a highly significant within-group improvement in the deep squat performance (VRT = *p* < 0.001, CI = 0.215 to 0.301, ES = 1.77) (CRT = *p* < 0.001, CI = 0.111 to 0.196, ES = 1.10). For vertical jump performance, there was a significant time by group interaction (*p* = 0.034, η_*p*_^2^ = 0.189). Simple effects analyses revealed significant post-experiment increases in the VRT (*p* < 0.001, CI = 3.264 to 5.704, ES = 1.13) and CRT (*p* < 0.001, CI = 1.382 to 3.822, ES = 0.75), but no significant between-group post-experiment difference (*p* = 0.069, ES = 0.78).

**TABLE 3:** Comparison of strength and jump performance assessment for within-and between-group.

	VRT			CRT		
	Pre	Post	Δ (Δ%)	Pre	Post	Δ (%)
Bench Press (kg/BW)	1.13 ± 0.09	1.30 ± 0.10^a^ ^c^	14.32	1.12 ± 0.07	1.19 ± 0.06^a^	6.21
Squat (kg/BW)	1.62 ± 0.17	1.88 ± 0.12^a^ ^b^	16.42	1.60 ± 0.16	1.76 ± 0.13^a^	9.89
CMJ (cm)	38.22 ± 4.20	42.71 ± 3.73^a^	12.02	37.58 ± 4.09	40.18 ± 2.65^a^	7.49

Note: BW: body weight; CMJ: countermovement jump.

^a^ Significantly different from baseline within group (*p* < 0.01).

^b^ Significantly different between the groups (*p* < 0.05).

^c^ Significantly different between groups (*p* < 0.01).

### Single punch performance

The mean values and changes in the single punch performance assessment are showed in Table 4. For the lead straight, no significant time by group interaction effect were observed for the maximum punch speed (*p* = 0.403, η_*p*_^2^ = 0.035). There was a significant main effect of time (*p* < 0.001, η_*p*_^2^ = 0.715). Both the VRT and CRT exhibited a significant within-group improvement in the punch speed (*p* < 0.001, CI = 0.667 to 1.221, ES = 1.53; 0.436 to 1.051, ES = 1.32, respectively). There was no significant different in the maximum punch seed between the VRT and CRT groups post-intervention (*p* = 0.403, ES = 0.40). For relative maximum punching force, there was a significant time by group interaction (*p* < 0.001, η_*p*_^2^ = 0.740); simple effects tests showed that post-experiment relative maximum punching force was significantly higher within the VRT (*p* < 0.001, CI = 0.059 to 0.087, ES = 1.17) but not within the CRT (*p* = 1.001, CI = −0.014 to 0.014, ES = 0.20), and that there was not a significant between-groups post-experiment difference (*p* = 0.133, ES = 0.35). Relative maximum punching power showed a significant time by group interaction effect (*p* = 0.030, η_*p*_^2^ = 0.215). Both groups exhibited a significant within-group improvement in the relative maximum punching power (VRT = *p* < 0.001, CI = 0.898 to 1.726, ES = 1.47) (CRT = *p* = 0.004, CI = 0.242 to 1.070, ES = 0.58). No significant post-experiment between-groups difference (*p* = 0.078, CI = −0.085 to 1.479, ES = 0.80).

**TABLE 4:** Comparison of single punch performance assessment for within-and between-group.

	VRT			CRT		
	Pre	Post	Δ(%)	Pre	Post	Δ(%)
LS-punch Speed (m/s)	7.40 ± 0.59	8.46 ± 0.78^b^	14.59	7.34 ± 0.61	8.17 ± 0.65^b^	11.58
LS-punch force (kg/BW)	1.11 ± 0.06	1.18 ± 0.06^b^	6.59	1.11 ± 0.15	1.14 ± 0.15	0.01
LS-punch power (w/BW)	8.30 ± 0.92	9.61 ± 0.86^b^	16.26	8.26 ± 1.32	8.91 ± 0.90^b^	9.23
RS-punch speed (m/s)	7.85 ± 1.05	9.25 ± 0.96^b^	18.87	7.96 ± 1.09	8.89 ± 0.85^b^	13.41
RS-punch force (kg/BW)	1.22 ± 0.07	1.31 ± 0.08^b^	7.34	1.21 ± 0.11	1.25 ± 0.07^a^	3.09
RS-punch power (w/BW)	10.22 ± 0.81	11.83 ± 0.98^b^	15.80	10.41 ± 1.92	11.21 ± 1.87^b^	8.35

Note: LS: lead straight; RS: rear straight; BW: body weight.

^a^ Significantly different from baseline within group (*p* < 0.05).

^b^ Significantly different from baseline within group (*p* < 0.01).

For the rear straight, no significant time by group interaction effect were observed for the maximum punch speed (*p* = 0.316, η_*p*_^2^ = 0.050). There was a significant main effect of time (*p* < 0.001, η_*p*_^2^ = 0.578). Both groups exhibited a significant within-group improvement in the maximum punch speed (VRT = *p* < 0.001, CI = 0.701 to 1.632, ES = 1.39) (CRT = 0.412 to 1.157, ES = 0.95). No significant difference between groups were found post-intervention (*p* = 0.316, ES = 0.40). Relative maximum punch force showed a significant time by group interaction effect (*p* = 0.005, η_*p*_^2^ = 0.334); simple effects tests showed significant post-experiment increases in the VRT (*p* < 0.001, CI = 0.063 to 0.115, ES = 1.20) and CRT (*p* = 0.013, CI = 0.008 to 0.059, ES = 0.43). There was not a significant between-groups post-experiment difference (*p* = 0.081, ES = 0.80). Relative maximum punching power showed a significant time by group interaction effect (*p* = 0.010, η_*p*_^2^ = 0.287). Both groups exhibited a highly significant within-group improvement in the maximum punching power (VRT = *p* < 0.001, CI = 1.190 to 2.027, ES = 1.79) (CRT = *p* = 0.001, CI = 0.384 to 1.221, ES = 0.42). No significant difference between-groups were found post-experiment (*p* = 0.341, ES = 0.42).

### 10s continuous punch performance

The mean values and changes in the 10s continuous punch performance assessment is showed in Table 5. For NP, there was a significant time by group interaction effect (*p* = 0.048, η_*p*_^2^ = 0.182). Both groups exhibited a highly significant within-group improvement in the VRT (*p* < 0.001, CI = 3.071 to 8.929, ES = 1.65) and the CRT (*p* < 0.001, CI = 7.253 to 13.110, ES = 0.81). No significant difference between-groups were observed post-intervention (*p* = 0.210, ES = 0.55). For APS, no significant time by group interaction effect were observed (*p* = 0.250, η_*p*_^2^ = 0.066). There was a significant main effect of time (*p* = 0.003, η_p_^2^ = 0.372). Both groups exhibited a significant within-group improvement in VRT = *p* = 0.003, CI = 0.180 to 0.735, ES = 0.85) and CRT (*p* = 0.005, CI = −0.133 to −0.461, ES = 0.45). There was no significant difference between the VRT and CRT groups post-intervention (*p* = 0.252, ES = 0.41). For CPF, no significant time by group interaction effect were found (*p* = 0.104, η_*p*_^2^ = 0.126). There was a significant main effect for time (*p* < 0.001, η_*p*_^2^ = 0.469). Both groups exhibited a significant within-group improvement in the VRT (*p* < 0.001, CI = 1.680 to 4.996, ES = 0.52) and CRT (*p* < 0.001, CI = 0.973 to 2.239, ES = 0.34). There was no significant difference between the VRT and CRT groups in the punch force post-intervention (*p* = 0.112, ES = 0.45). For CPP, there was a significant time by group interaction effect (*p* = 0.018, η_*p*_^2^ = 0.250); simple effects tests showed a significant post-experiment increase in the VRT (*p* < 0.001, CI = 3.071 to 8.929, ES = 0.78) and the CRT (*p* < 0.001, CI = 7.253 to 13.110, ES = 0.32). No significant difference between-groups were found post-intervention (*p* = 0.210, ES = 0.49).

**TABLE 5:** Comparison of 10s continuous punch performance assessment for within-and between-group.

Items	VRT			CRT		
	Pre	Post	Δ (%)	Pre	Post	Δ (%)
NP (times)	52.00 ± 6.47	62.18 ± 5.86^a^	20.38	52.82 ± 8.33	58.82 ± 6.31^a^	12.42
APS (m/s)	7.53 ± 0.67	8.15 ± 0.79^a^	8.50	7.57 ± 0.78	7.87 ± 0.54^a^	4.44
CPF (kg/BW)	53.22 ± 7.62	57.91 ± 10.19^a^	8.65	52.26 ± 6.30	54.24 ± 5.21^a^	4.11
CPP (w/BW)	398.49 ± 80.10	475.67 ± 115.10^a^	19.18	401.22 ± 72.14	426.36 ± 85.24^a^	6.27

Note: NP: number of punches; APS, average punch speed; CPF: cumulative punch force; CPP: cumulative; BW: body weight.

^a^ Significant difference from baseline within group (*p* < 0.01).

### 30s continuous punch performance

The mean values and changes in the 30s continuous punch performance assessment are showed in Table 6. For NP, two-factor repeated-measures ANOVA revealed a significant time main effect (*p* < 0.001, η_p_^2^ = 0.718), no group main effect (*p* = 0.417, η_p_^2^ = 0.033), and no significant time by group interaction effect (*p* = 0.155, η_p_^2^ = 0.098); *post hoc* tests showed significant post-experiment increases in both the VRT and CRT (*p* < 0.001, CI = 7.301 to 13.335; −13.335 to −7.301); independent samples t-tests showed no significant between-groups difference (*p* = 0.155, CI = −10.307 to 1.761, ES = 0.47). For APS, there was no significant time by group interaction effect (*p* = 0.255, η_p_^2^ = 0.064), no significant time main effect (*p* = 0.145, η_p_^2^ = 0.103), and no significant group main effect (*p* = 0.585, η_p_^2^ = 0.015); independent samples t-test showed no significant between-group post-experiment difference (*p* = 0.255, CI = −0.956 to 0.268, ES = 0.36). For CPF, there was a significant time main effect (*p* = 0.001, η_p_^2^ = 0.450, CI = −5.714 to 5.714), no significant main group effect (F = 0.085, *p* = 0.773, η_p_^2^ = 0.004), and no significant time by group interaction effect (*p* = 0.632, η_p_^2^ = 0.012); *post hoc* test showed significant post-experiment increases in both the VRT and the CRT (*p* = 0.001, CI = 1.827 to 5.714; −5.714 to −1.827, respectively). Independent samples t-test showed no significant between-group difference (*p* = 0.632, CI = −4.792 to 2.981, ES = 0.16). For CPP, there was a significant time by group interaction effect (*p* = 0.003, η_p_^2^ = 0.359); simple effects tests showed significant post-experiment increases in relative cumulative punching power in the VRT (*p* < 0.001, CI = −35.752 to −13.262) and CRT (*p* < 0.001, CI = −61.267 to −38.777), and no significant between group post-experiment difference (*p* = 0.391, CI = −94.843 to 38.688, ES = 0.37).

**TABLE 6:** Comparison of 30s continuous punch performance assessment for within-and between-group.

Items	VRT			CRT		
	Pre	Post	Δ (%)	Pre	Post	Δ (%)
NP (times)	136.64 ± 12.17	149.09 ± 15.81^a^	9.13	134.18 ± 12.76	142.36 ± 12.70	6.18
APS (m/s)	6.80 ± 0.71	7.20 ± 0.63	6.61	6.86 ± 0.57	7.02 ± 0.32	1.29
CPF (kg/BW)	51.84 ± 8.10	56.06 ± 9.38^a^	8.51	51.37 ± 5.25	54.69 ± 7.48	6.21
CPP (w/BW)	353.91 ± 82.40	403.93 ± 86.25	14.78	351.35 ± 58.59	375.85 ± 61.89	7.26

Note: NP: number of punches; APS: average punch speed; CPF: cumulative punch force; CPP: cumulative.

^a^ Significantly different from baseline within group (*p* < 0.01).

## Discussion

This study compared the effects of VRT and CRT, within a complex training program, on muscle strength, jumping ability, and boxing performance in elite boxers. The results showed that both VRT and CRT significantly improved the maximum strength, vertical jump, and punch performance of elite boxers, but VRT was more effective in improving maximum strength.

### Strength performance

The results herein show that post-intervention relative strength, based on bench press and squat, improved significantly more in the VRT compared with the CRT. The bench press (*r* = 0.76) (Loturco et al., 2021), squat (*r* = 0.79) (Loturco et al., 2021), and CMJ (*r* = 0.72) (Loturco et al., 2016) exercises were significantly associated with the peak power of straight anterior and straight posterior punches in boxers. By combining resistance training with plyometric training in complex training, CRT leverages the post-activation potentiation effect of muscle contraction, optimizes the benefits of maximum strength and explosive strength, and significantly reduces the risk of injury (Duthie et al., 2002). On the basis of traditional constant resistance, VRT combines with elastic bands or iron chains to change the external load throughout the range of motion. This method can significantly enhance athletes’ strength during the concentric phase and lead to greater power output. VRT is superior to CRT in enhancing maximum strength. This is primarily because the gradually increasing load during the concentric phase of VRT can adjust to the force generation capacity of muscles at various joints, thereby optimizing strength performance (Wallace et al., 2018). When the bench press and squat reach their lowest position, the external load exerts the maximum moment arm at the shoulder, elbow, hip, and knee, respectively. At this time, each joint needs to generate a large internal moment to resist the maximum external moment, while the skeletal muscle has the lowest leverage efficiency at this position. The muscle faces the greatest difficulty in generating force, a phenomenon known as the “sticky point” (Kompf and Arandjelović, 2017). With the extension of the joint, the external torque decreases gradually. The muscle reaches the optimal length of contraction, and the force generation ability of the muscle increases gradually. The joint can bear the maximum external load when it is fully extended (Shi et al., 2022b). VRT incorporates additional resistance (i.e., elastic bands and chains), which leads to increasing and decreasing resistance during concentric and eccentric phases, thereby overcoming the “sticking zone,” or the range of motion where deceleration occurs due to skeletal muscle weaknesses during in resistance training, which compensates for the lack of muscle stimulation by CRT and promotes muscle strength growth (Kompf and Arandjelović, 2016). However, the VRT showed greater improvement in strength performance than the CRT. This may be attributed to the additional training effect of VRT, which maintained these athletes’ muscle tension at consistently higher levels across changes in joint angles throughout the exercise. Findley et al. have also indicated that the effects of strength training interventions will be optimized if the maximum exerted force is matched across joint angles (Findley, 2004). Thus, VRT is more effective than CRT alone in improving both upper and lower extremity strength and explosive power, which may help establish a foundation for increased punching force. Andersen’s Meta-analysis (Andersen et al., 2022) showed that there was no significant difference in the maximum strength and explosive power of the upper and lower limbs between VRT and CRT. However, subgroup analysis found that: training period (number of weeks) and number of repetitions per set significantly moderated the effects of VRT vs. CRT on maximal lower body strength. Significant and small-sized effects on maximal lower body strength were observed in favor of TRT for 8–12 repetitions per set, but not for <8 repetitions per set. For training period, no significant effects were found for the comparison of VRT versus TRT.

The results of a meta-analysis (Shi et al., 2022a) and an experimental study (Shi et al., 2022b) on CMJ are similar to those of the present study, indicating that VRT does not differ significantly from CRT in enhancing the training effect of explosive power. Based on the load characteristics of VRT, the design method with the same intensity results in a higher intensity at the top of the motion range. Although overloading can effectively enhance the peak strength of the eutectic phase (Wallace et al., 2006), it can also result in a reduction in the rate of action (Saeterbakken et al., 2016; Stevenson et al., 2015). This significant decrease in speed can consequently lead to a decline in power output (Swinton et al., 2011). Therefore, the overload characteristics of VRT may negatively affect the performance of explosive power when speed is the goal (Shi et al., 2022a). Some studies (Israetel et al., 2010) have pointed out that overloading can significantly increase velocity, force, and power output at the end of the centripetal phase. This may be related to the significant increase in strength at the end of the centripetal phase that outpaced the effect of the decrease in velocity on burst power.

### Boxing performance

#### Single punch performance

The findings of the present study revealed that the maximum single punch speed, relative maximum punch force, and relative maximum punch power were increased in both groups. The micro-mechanism of the CRT effect is its enhancement of motor unit excitability, which improves recruitment levels. It also regulates myosin light chain phosphorylation, making myofilaments more sensitive to calcium ions, while reducing presynaptic inhibition, creating conditions for enhancing subsequent explosive power output (Hodgson et al., 2005). At the macroscopic level, CRT facilitates more comprehensive development of all components of the tension–velocity curve, contributing to notable output power increases (Berriel et al., 2022). A heavier load enhances the high-power portion of the curve, while a lighter load and higher movement speed affects the high-speed region of the curve (Haff and Nimphius, 2012). During the training process, whether high load resistance or complex training, athletes are always asked to complete each movement as quickly as possible. It is well known that movement speed is the key element in training effectiveness under equivalent load conditions. Therefore, CRT can induce more comprehensive adaptive changes across the entire tension–velocity curve, making it more effective than either high-load or explosive training in isolation (Berriel et al., 2022).

In this study, results showed that the VRT exhibited greater improvement in relative maximum punch force, punch power, and punch speed compared with the CRT. This may be because the variable resistance loads change the form of tension loading during CRT, allowing resistance adaption to leverage changes and thus reducing the mechanical disadvantages of specific angles on movement speed and providing compensatory acceleration (Frost et al., 2010). Furthermore, VRT produces a nonstable form that helps athletes enhance their capacity to tackle heavier loads, thus stimulating the neuromuscular system beyond CRT alone (Zimmermann et al., 2020). Variable resistance training also allows greater velocity during the first half of the movement, due to its lighter load, possibly promoting neuromuscular activation. Stronger neuromuscular activation enhances post-activation performance, resulting in increased movement speed (Stevenson et al., 2010). It is thus evident that VRT can improve boxers’ relative maximum punch force and power, which is closely related to the neural control and post-activation performance enhancement effect observed in high-load resistance training.

#### Continuous punch performance

The 10s and 30s punch performances can reflect the repeated attack capacity of boxers. It has been reported that the number of punches were high for winners than losers in competition (Davis et al., 2013). Our findings showed that VRT and CRT can effectively improve the continuous punch performance. This may be because the complex training provides effective stimulation and activation of the neural and muscular systems (Scott et al., 2017). By shortening the interval between high strength resistance and super isometric training, and by completing the “double complex set” conversion in the first 15s after the adjustment, phosphate system mobilization is repeated in the training unit, gradually increasing the proportion of glycolytic energy supply. Many studies have indicated that during high-intensity exercise, the proportion of supply from the glycolytic and aerobic systems increased with exercise duration (Gibala et al., 2012). In addition, as the duration of the muscle contraction increases during high-intensity exercise, the involved motor units become fatigued. To maintain exercise intensity, higher-order motor units belonging to type II muscle fibers, are mobilized to participate in contraction. This can mobilize more phosphocreatine and lactate involved in energy supply (Haff, 2016).

In contrast, traditional high-load resistance training is typically biased towards the phosphate energy supply system because there is sufficient recovery after each set. Because it takes about 4 minutes for recovery from phosphagen fatigue to a level that will not affect the next training set, sufficient intervals can replenish phosphate metabolic reserve, and facilitate mobilization and stimulation of the phosphate metabolic system (Morales-Alamo et al., 2015). If the athlete’s phosphate reserves are not fully recovered before the start of the next exercise, the proportion of the phosphate system involved in energy supply decreases. In the present study, the interval between sets was 2 min, which is insufficient for full phosphate system recovery. Because the proportion of the phosphate system’s energy supply decreases with increasing repetitions, insufficient recovery of the phosphate metasystem inevitably leads athletes to mobilize energy supplies from the glycolytic system earlier (Girard et al., 2011). VRT offers additional loads throughout the entire range of motion during exercise, which induces greater total work and muscle activation when compared to CRT (Arazi et al., 2020). Although there were the same number of repetitions in both groups, there was higher intensity and more energy consumption in the VRT. Therefore, the VRT showed greater improvement in strength and punch performance than the CRT.

## Conclusion

The main findings of the present study demonstrated that both VRT and CRT can enhance maximal strength and vertical jump performance in boxers. VRT is more effective for improving upper and lower extremity muscle strength compared with CRT. In additional, VRT will enhance at a greater magnitude than the CRT in single and continuous punch performance. Future studies should consider extending the duration of the training intervention to assess the long-term effects of VRT and CRT on muscle strength and exercise performance, as well as their sustainability. Additionally, research should examine athletes across various training levels, genders, and age groups to validate the generalizability and adaptability of these training methods. Furthermore, future investigations should explore the effects of different training intensities and volumes on athletic performance, as well as how training programs can be customized to optimize training outcomes. It is also advisable to conduct more comprehensive studies on the effects of VRT and CRT within complex training regimens, including an analysis of changes in muscle activation patterns, motor unit recruitment strategies, and muscle fiber types.

## Limitations

This study has several limitations, including a relatively small sample size and a sample selection that is specific to elite female boxers, which may restrict the generalizability of the findings. Additionally, the study primarily focused on measures of strength and boxing performance, neglecting to assess physiological and biochemical factors that could offer further insights into training adaptations.

## Practical implications

1. Based on the test results, it can be concluded that VRT is effective in enhancing the explosive power of both the upper and lower limbs of boxers. Furthermore, VRT has also been found to enhance the boxers’ specific hitting ability. Therefore, strength and conditioning coaches should consider incorporating VRT into their training plans to enhance muscle strength and improve punch performance in athletes.
2. The findings of this study suggest that elite amateur boxers possess a solid training foundation and can consider adopting this training method. This method not only offers a different approach to training, making it more enjoyable, but also effectively enhances training efficiency.
3. This training method has been widely confirmed in other fields, but there are few studies on its application in the field of boxing, and this study confirmed the effectiveness and reliability of this training method through training intervention, and it is relatively safe for boxers.

## Data Availability

The raw data supporting the conclusions of this article will be made available by the authors, without undue reservation.
